# Long Non-Coding RNA LOC339059 Attenuates IL-6/STAT3-Signaling-Mediated PDL1 Expression and Macrophage M2 Polarization by Interacting with c-Myc in Gastric Cancer

**DOI:** 10.3390/cancers15225313

**Published:** 2023-11-07

**Authors:** Haibo Han, Guangyu Ding, Shanshan Wang, Junling Meng, Yunwei Lv, Wei Yang, Hong Zhang, Xianzi Wen, Wei Zhao

**Affiliations:** 1 Department of Clinical Laboratory, Key Laboratory of Carcinogenesis and Translational Research (Ministry of Education), Peking University Cancer Hospital & Institute, Beijing 100142, China; haibohan@bjmu.edu.cn (H.H.); shanshanwang@bjcancer.org.cn (S.W.);; 2 Department of Gastric Surgery, Zhejiang Cancer Hospital, Hangzhou 310022, China; dinggy@zjcc.org.cn

**Keywords:** LOC339059, c-Myc, IL-6, gastric cancer, PDL1, macrophage polarization

## Abstract

**Simple Summary:**

In this study, we analyzed the role of LOC339059 in PDL1 expression and M2 polarization, which is mediated via the inhibition of IL-6/STAT3 signaling through interaction with c-Myc. Based on a retrospective cohort study, LOC339059 expression was determined to be down-regulated in primary gastric cancer tissues compared with adjacent tissues, and its low expression predicts a poor survival time after surgery. Furthermore, its level of expression was found to be correlated with the expression of some immune response genes. Functionally, LOC339059 acts as a tumor suppressor that can suppress malignant cell phenotypes, cell PDL1 expression, and macrophage M2 polarization. Mechanically, nucleus-localized LOC339059 interacts with c-Myc, competitively inhibiting the latter’s ability to promote IL-6 transcription, thereby reducing IL-6/STAT3-mediated PDL1 expression and M2 polarization. Our results establish c-Myc as a pivotal factor at the crossroads of the LOC339059-mediated IL-6/STAT3-dependent regulation of the immune response.

**Abstract:**

**Background:** Long non-coding RNA (lncRNA) was identified as a novel diagnostic biomarker in gastric cancer (GC). However, the functions of lncRNAs in immuno-microenvironments have not been comprehensively explored. In this study, we explored a critical lncRNA, LOC339059, that can predict the clinical prognosis in GC related to the modulation of PD-L1 and determined its influence upon macrophage polarization via the IL-6/STAT3 pathway. **Methods:** To date, accumulating evidence has demonstrated that the dysregulation of LOC339059 plays an important role in the pathological processes of GC. It acts as a tumor suppressor, regulating GC cell proliferation, migration, invasion, tumorigenesis, and metastasis. A flow cytometry assay showed that the loss of LOC339059 enhanced PDL1 expression and M2 macrophage polarization. RNA sequencing, RNA pull-down, RNA immunoprecipitation, Chip-PCR, and a luciferase reporter assay revealed the pivotal role of signaling alternation between LOC339059 and c-Myc. **Results:** A lower level of LOC339059 RNA was found in primary GC tissues compared to adjacent tissues, and such a lower level is associated with a poorer survival period (2.5 years) after surgery in patient cohorts. Moreover, we determined important immunological molecular biomarkers. We found that LOC339059 expression was correlated with PD-L1, CTLA4, CD206, and CD204, but not with TIM3, FOXP3, CD3, C33, CD64, or CD80, in a total of 146 GC RNA samples. The gain of LOC339059 in SGC7901 and AGS inhibited biological characteristics of malignancy, such as proliferation, migration, invasion, tumorigenesis, and metastasis. Furthermore, our data gathered following the co-culture of THP-1 and U937 with genomic GC cells indicate that LOC339059 led to a reduction in the macrophage cell ratio, in terms of CD68^+^/CD206^+^, to 1/6, whereas the selective knockdown of LOC339059 promoted the abovementioned malignant cell phenotypes, suggesting that it has a tumor-suppressing role in GC. RNA-Seq analyses showed that the gain of LOC339059 repressed the expression of the interleukin family, especially IL-6/STAT3 signaling. The rescue of IL-6 in LOC339059-overexpressing cells reverted the inhibitory effects of the gain of LOC339059 on malignant cell phenotypes. Our experiments verified that the interaction between LOC339059 and c-Myc resulted in less c-Myc binding to the IL-6 promoter, leading to the inactivation of IL-6 transcription. **Conclusions:** Our results establish that LOC339059 acts as a tumor suppressor in GC by competitively inhibiting c-Myc, resulting in diminished IL-6/STAT3-signaling-mediated PDL1 expression and macrophage M2 polarization.

## 1. Introduction

Gastric cancer (GC) is the third most common cancer and the third leading cause of cancer mortality in China [1]. More than 40% of new and fatal cases of GC occur in China [2]. Most patients with GC have been diagnosed at an advanced stage, with a poor prognosis, limited chemotherapy responses, and a low five-year survival rate [3].

In the tumor microenvironment, macrophages are the most abundant immune cells, named tumor-associated macrophages (TAMs) [4]. Macrophages have two distinct functional phenotypes: M1 (pro-inflammatory, classically activated macrophage) and M2 (anti-inflammatory, alternatively activated macrophages) subtypes [5,6]. M1 macrophages can destroy cancer cells by phagocytosis, whereas M2 macrophages, characterized by the expression of CD163 or CD206 on their cell surface, promote tissue repair and angiogenesis and favor tumor progression by producing anti-inflammatory cytokines [7]. The polarization of macrophages can be repolarized by several essential signaling pathways [8], including the IL-6R/STAT3 signaling pathway [9]. Therefore, decreasing M2-phenotype macrophages is a promising strategy for future cancer immunotherapy. It has been reported that IL-6 is a critical factor for inducing polarization toward the M2 phenotype. IL-6 derived from prostate epithelial cells promotes the progression of prostate cancer by inducing the M2 polarization of THP-1-derived macrophages [10]. Increased IL-6 secretion plays a vital role in developing colorectal cancer by increasing M2 polarization in mouse models [11]. In addition to the regulation of macrophage polarization, IL-6 can also protect tumors from immunotherapy with immune checkpoint inhibitors (ICIs) [12].

Non-coding RNAs (lncRNAs) are a class of transcripts that are more than 200 nucleotides in length and that are not translated into proteins [13]. Notably, lncRNAs have been reported to be deregulated in many human diseases, including tumors. Recent studies have reported that the abnormal expression of various lncRNAs is related to the diagnosis, prognosis, occurrence, and development of GC and the immune-microenvironment-related progression of GC [14]. LncRNAs regulate the immune microenvironment by impacting tumor-infiltrating immune cells or immune checkpoint molecules. Pyroptosis-related four-lncRNA can be used to effectively predict GC patients’ prognosis and the immune microenvironment [15]. Ferroptosis-related lncRNAs indirectly impaired the activation of CD4^+^ T cells in GC [16]. Ding et al. constructed a prognostic model based on immune-related lncRNAs (IRLs), which can predict the overall survival and ICI responses of patients with GC [17]. Huang et al. reported 23 m6A-related lncRNAs related to tumor-infiltrating immune cells, the expression of programmed death-1 (PD-1), and cytotoxic T-lymphocyte-associated protein 4 (CTLA4) [18]. Wang et al. reported eighteen immune-related lncRNA pair (IRLP) signatures associated with cancer-associated fibroblasts; macrophage M2 infiltration; and PD-L1, CTLA4, LAG3 (Lymphocyte Activation Gene-3), and HLA expression in GC [19]. Wang et al. reported the expression profiles of 15 m6A-related lncRNAs linked to the immune score, stromal score, and ESTIMATE score in GC [20]. The above results indicate that lncRNAs are central to the immune microenvironment and checkpoints of cancer [14,21,22]. Recently, based on machine learning, the aberrant expression of the lncRNA LOC339059 was first identified as a potential diagnostic biomarker for papillary thyroid carcinoma [23,24]. However, its role in gastric cancer is still unclear. In this study, we aimed to explore the expression signature of LOC339059 to predict the OS of patients with gastric cancer and the effect of LOC339059 on the malignant phenotypes of gastric cancer cells. We also investigated the molecular mechanism by which LOC339059, as a tumor suppressor, inhibits PDL1 and macrophage M2 polarization by modulating the activity of the IL-6/STAT3 axis in gastric cancer cells. Our findings may help unravel the role of LOC339059 in the immunosuppressive microenvironment, thereby providing potential targets for treating gastric cancer. 

## 2. Materials and Methods

### 2.1. Established Cancer and Isogenic Cell Lines

Established cell lines, namely, SGC-7901, AGS, and BGC-823, were maintained in RPMI 1640 medium supplemented with 10% FBS (Newzerum, Christchurch, New Zealand, # FBS-E500) and 1% penicillin–streptomycin. We authenticated the human monocytic leukemia cell line THP1 and human acute promyelocytic leukemia cell line U937 using STR DNA profiling (HuaKeJianLian, Beijing, China).

LOC339059 overexpression and knockdown cell lines were established using LOC339059 (NR_120387) and shRNA (targeting RNA sequence at 474–495; 536–561 sites) lentiviral expression plasmids. The lentiviral shuttle vectors were transfected into HEK 293FT cells with the helper plasmids PLP1, PLP2, and pLP/VSVG to generate lentiviral particles that were used to infect cancer cells, which were then screened using 20 μg/mL blasticidine. C-Myc knockdown cells were generated by transfecting cells with genOFF h-Myc (Ribobio, Guangzhou, China, #SIGS0003660-1) using Lipofectamine 2000 (Invitrogen, Waltham, MA, USA #11668019), and in vitro experiments were performed after 48 h of transfection.

### 2.2. Clinical Specimens

Primary GC specimens with specific inclusion/exclusion criteria for patients were obtained as previously described [25]. This study was approved by the Research and Ethical Committee of Peking University Cancer Hospital (2017KT79).

### 2.3. Total RNA Extraction, Nuclear and Cytoplasmic RNA Fractionation, Real-Time qPCR, and RNA Sequencing Analysis

Total RNA was isolated using Rneasy mini kits (QIAGEN). cDNA was synthesized using 2 μg of total RNA using random primers and Moloney murine leukemia virus reverse transcriptase (M-MLV RT) (Invitrogen, Carlsbad, CA, USA, #28025013). Q-PCR was carried out using SYBR Green PCR Mix (Applied Biosystems, Waltham, CA, USA, # A25742) on an ABI 7500 system (Applied Biosystems). The relative gene expression level was calculated using the 2^−ΔCt^ method, where ΔCt = Ct (target gene) − Ct (GAPDH). Q-PCR data are represented as the mean ± SD from three independent triplicate experiments. RNA sequencing (RNA-seq) was performed using Novogene (Beijing, China). 

### 2.4. RNA In Situ Hybridization (ISH)

The ISH assay was conducted using an in situ hybridization kit (Boster Biological Technology Co., Ltd., Wuhan, China) with four mixed LOC339059 probes labeled with digoxin. Briefly, the cell slides were fixed with 4% formaldehyde for 5 min and permeabilized with 0.5% Triton X-100 for 10 min. After blocking with pre-hybridization buffer for 30 min at 37 °C, the slides were incubated overnight with a 0.5 μg/mL probe in a hybridization buffer at 37 °C. After washing with saline sodium citrate (SSC) buffer, the cell slides were then incubated with an anti-digoxin monoclonal antibody labeled with fluorescein (1:100 dilution, Jackson ImmunoResearch, West Grove, PA, USA) for 30 min at 37 °C, followed by counterstaining with DAPI for 5 min. Fluorescence was detected using a fluorescent microscope (Dmi8, Leica, Weitzlar, Germany). The frozen tissue sections were stained according to the standard manufacturer’s protocol. 

### 2.5. Cell Viability

A total of 5 × 10^3^ cells/well were seeded into 96-well plates (*n* = 5 per group). Cell viability was evaluated using Cell Counting Kit-8 (CCK-8; Dojindo, Kumamoto, Japan, #CK04) at the defined time according to the manufacturer’s instructions. Briefly, 10 μL of CCK-8 reagent was added to each well and incubated for one hour, and then the absorbance was measured at 450 nm with a microplate reader (iMark, Bio-Rad Laboratories, Hercules, CA, USA).

### 2.6. Plate Colony Formation Assay

Cells (10^3^/well) were seeded into 6-well plates in triplicate and cultured for another 1 w. The surviving colonies (more than 10 cells) were counted after fixation with 4% formaldehyde for 5 min and staining with 1% crystal violet for 10 min at room temperature. 

### 2.7. Transwell Migration and Invasion Assay

Cells (1 × 10^5^) pretreated with 10 μg/mL mitomycin-C (Sigma, St. Louis, MO, USA) for 1 h at 37 °C were suspended in 100 μL of RPMI 1640 medium with 1% FBS and seeded into the upper chamber of a Transwell (Corning, NY, USA, #3422) either without (for migration assays) or coated with (for invasion experiments) 50 μL of Matrigel™ Basement Membrane Matrix (1:5 in RPMI 1640). Five hundred microliters of RPMI 1640 containing 10% FBS as a chemoattractant was added to the lower chamber. Twenty-four hours later, the cells were fixed with 4% formaldehyde for 5 min and stained with 0.1% crystal violet for 10 min at room temperature. After wiping the cells inside the membrane, the number of migrated or invasive cells was determined by taking a photograph and counting the cells in four randomly selected microscopic fields.

### 2.8. In Vivo Tumor Growth and Metastasis

The tumor growth and metastatic characteristics were measured using a modified chicken embryo chorioallantoic membrane (CAM) assay. Briefly, 10-day-old SPF white leghorn chicken eggs (Beijing Merial Vital Laboratory Animal Technology Co., Ltd., Beijing, China) were randomized into groups (*n* = 5 per group). After sterilization with 75% ethanol, a square window in the shell was opened under aseptic conditions. Cells were pre-labeled with the cell tracker CM-DiI (red fluorescent dye) in 5% glucose for 15 min at 37 °C, and then a total of 5 × 10^6^ cells in 50 μL of PBS were used to inoculate the surface of each CAM. Eggs were returned to a humidified 37 °C incubator for an additional 9 d. The tumors that grew on the CAMs of eggs were dissected and weighed by an investigator blinded to the group allocation. To track the metastatic tumor cells, the lungs of the chicken embryos were isolated, flattened between two slides, and evaluated under a fluorescence microscope (Leica, Germany). 

Six-week-old NOD/SCID male mice (NOD. CB17-prkdcscid/NcrCrl, Vital River, Beijing, China) were randomized into groups (*n* = 5 per group). Tumor growth was monitored weekly until the tumor size exceeded 1 cm, defined as an endpoint. The mice were then sacrificed, and the tumors were stripped for weighing. The animal studies were approved by the institutional guidelines of the Peking University Cancer Hospital Animal Care Committee (EAEC_2018-21).

### 2.9. Flow Cytometer Analysis

M0 and M2 macrophages were identified using anti-CD68 and anti-CD206 antibodies, respectively. Cells were stained with anti-CD68 FITC and anti-CD206 PE (eBioscience, San Diego, CA, USA). After incubation at 4 °C in the dark for 30 min, the cells were washed three times with PBS, resuspended in PBS supplemented with 1% FBS, and analyzed using flow cytometry (Beckman Coulter, Brea, CA, USA). 

### 2.10. Immunoblotting

Cells were lysed using a radioimmunoprecipitation assay (RIPA) buffer containing a protease and phosphatase inhibitor cocktail (Roche, Basel, Switzerland). The protein concentration was quantified using a Bio-Rad protein assay kit (Bio-Rad Laboratories, Hercules, CA, USA). Equal amounts of protein from each sample (20 μg) were separated on 10% SDS-PAGE gels and transferred to polyvinylidene difluoride membranes (Merck Millipore, Billerica, MA, USA). Proteins were detected using the following specific antibodies: PDL1 (Abcam, Cambridge, UK, #ab279292), GAPDH (Santa Cruz Biotechnology, Dallas, TX, USA, #sc-4772), p-STAT3 (Tyr705; #9145), STAT3 (CST, #12640), IL-6 (Abcam, #ab9324), c-Myc (Abcam, #ab32072), and HRP-conjugated secondary antibodies (Jackson ImmunoResearch). Images were visualized using ECL detection reagents (Millipore, Billerica, MA, USA) and captured using AI600 version 1.2.0 on an Amersham Imager 600 (GE Healthcare, Chicago, IL, USA).

### 2.11. RNA Pull-Down, RNA-Binding Protein Immunoprecipitation (RIP), and Chromatin Immunoprecipitation (ChIP) Assay

For the in vivo pull-down assay, lncRNA-6×MS2bs plasmids containing six repeat MS2-binding-site RNA sequences and MS2 expression plasmids with Flag tags were co-transfected into cells, and then the complexes were isolated using anti-Flag-conjugated magnetic beads (Sigma, Louis, MO, USA). The proteins were precipitated using pull-down assays and analyzed using Western blotting using the defined antibodies. The RNA in the cell lysates was immunoprecipitated for the RIP experiments with 3 µg of antibody or nonspecific IgG. Immunoprecipitated RNAs were isolated using TRIzol LS reagent, followed by cDNA synthesis for q-PCR. A ChIP assay was performed using a SimpleChIP enzymatic chromatin IP kit (Cell Signaling Technology, Danvers, MA, USA). Purified DNA was subjected to immunoprecipitation with 3 µg of antibody and nonspecific IgG. The enriched DNA was purified and analyzed via q-PCR.

### 2.12. Promoter-Driven Reporter Gene Assay

Cells were seeded into 24-well plates and then co-transfected with 300 ng of pGL3-IL6-luc containing a 2 kbp promoter region, 26 ng of pRL-TK plasmid expressing Renilla luciferase, and 20 pmol of siRNAs using Lipofectamine 2000 (*n* = 4 per group). After 24 h, the firefly and Renilla luciferase activity levels in the cell lysates were determined using a dual-luciferase reporter assay kit (Promega Corporation, Madison, WI, USA) according to the manufacturer’s protocol. The firefly luciferase activity was normalized to that of the Renilla luciferase for each sample. The experiments were repeated three times with four replicates.

### 2.13. Statistical Analysis

The data were analyzed using SPSS 13.0 and GraphPad Prism 8 software. For the clinical cohort, 146 participants were recruited, resulting in more than 80% power and an alpha of 5%. A two-tailed χ^2^ test was conducted to evaluate the correlation between lncRNA and clinicopathological factors. Nonparametric testing was used to compare the LOC339059 expression in the GC and normal tissues. Overall survival (OS) was analyzed using the Kaplan–Meier method and log-rank test. The data for continuous variables with normal distributions (Kolmogorov–Smirnov test) and equal variance (F-test) between/within the groups are expressed as the mean  ±  SD from three independent experiments in triplicate (or with more replicates). We tested the statistical significance for two groups using a two-tailed Student’s *t*-test, one-way ANOVAs with Bonferroni post hoc tests for multiple comparisons, and two-way ANOVA for cell viability analysis based on the treatment and time course. A two-tailed test with *p*  <  0.05 was considered to indicate a statistically significant difference.

## 3. Results

### 3.1. LOC339059 Is Down-Regulated in Gastric Cancer, and Low Expression Predicts Poor Survival

**Figure 1:**
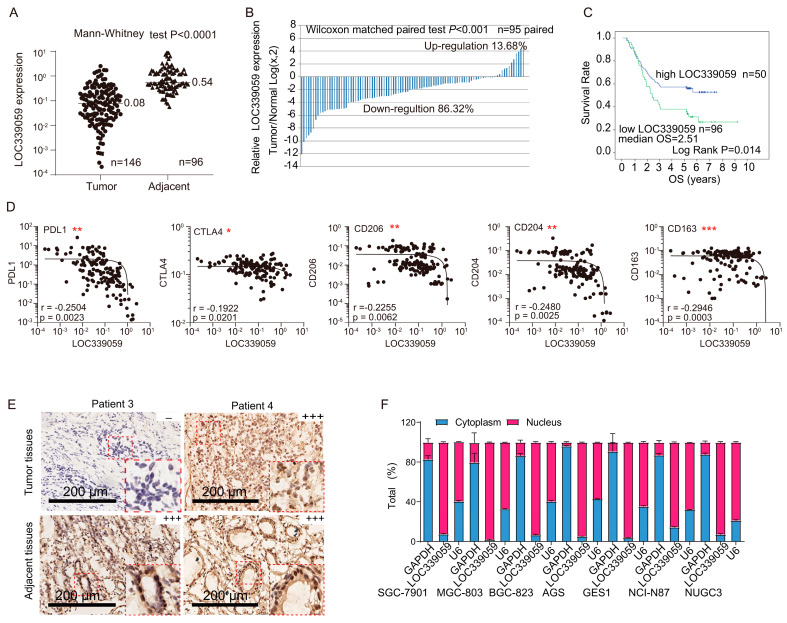
LOC339059 expression was down-regulated in gastric cancer tissues and was associated with the survival times of patients after surgery and the expression of PDL1; CTLA4; and the M2 macrophage markers CD206, CD204, and CD163. (**A**,**B**) The qRT-PCR assay confirmed that LOC339059 expression was down-regulated in gastric cancer tissues compared with adjacent tissues. (**C**) K-M survival analysis showed that low expression of LOC339059 in gastric cancer tissues was associated with the poor overall survival of patients (*p* = 0.014). (**D**) Linear correlation analysis indicated that LOC339059 expression was correlated with the expression of immune inhibitory molecules (PDL1 and CTLA4) and M2 macrophage markers (CD206, CD204, and CD163) in tumors. * *p* < 0.05; ** *p* < 0.01; *** *p* < 0.001. (**E**) In situ hybridization showed nuclear localization of LOC339059 in the tumor and adjacent tissues. − negative; +++ strong positive.(**F**) The relative expression ratio of LOC339059 in the nucleus and plasma in gastric cancer was analyzed via qRT-PCR.

Since we had previously identified the aberrant expression of LOC339059 in gastric cancer [25], we performed a quantitative PCR analysis of 146 gastric cancer tissues and 96 adjacent tissues to further analyze the LOC339059 RNA level and clinical features. Our results show that LOC339059 expression in gastric cancer was significantly lower than that in adjacent tissues (Figure 1A, *p* < 0.001). In 86.32% (82/95) of patients, LOC339059 was down-regulated in tumor tissues to a much greater extent than in adjacent tissues in all pairs of matched samples (Figure 1B, paired *t*-test, *p* < 0.001). The patients were divided into high- and low-expression groups according to the cutoff value of the lncRNA determined via the maximum Jordan index of the ROC curve. As shown in Figure 1C, the lower the LOC339059, the worse the OS of patients, whose median OS was only 2.51 months. 

### 3.2. LOC339059 Is Associated with the Expression of Immune Genes and Macrophage Biomarkers

To investigate whether the expression of LOC339059 is directly related to immune regulation, the profiling of immune checkpoint genes and molecular biomarkers was analyzed. We found that the expression of LOC339059 was negatively correlated with that of PDL1 and CTLA4 (Figure 1D) but not with the presence of TIM3. Furthermore, among the biomarkers that negatively correlated with LOC339059, CD206, CD204, and CD163 were validated, while CD3, CD8a, CD8b, FOXP3, NKG2D, CD33, CD64, CD86, and CD80 did not show significant correlations (Figure 1D and Figure S1). 

The subcellular localization of lncRNA is critical to its function [26]. Nuclear lncRNAs are involved in the modulation of transcriptional programs through chromatin interactions or the remodeling and establishment of the spatial organization of the nuclear compartment via scaffolding. By contrast, cytoplasmic lncRNAs mediate signal transduction pathways, modulate translational programs, and exert post-transcriptional control of gene expression [26]. Thus, we determined the subcellular location of LOC339059. The in situ hybridization (Figure 1E) of tissue samples and quantitative PCR analysis (Figure 1F) of plasma and nuclear RNA isolates from seven GC cell lines showed that LOC339059 was mainly distributed in the nucleus, indicating that it functioned in transcriptional regulation. 

### 3.3. Low LOC339059 Is Associated with Poor Differentiation of GC

Furthermore, we analyzed the relationship between the expression level of LOC339059 and the clinical characteristics of gastric cancer patients. As shown in Table 1, clinical data analysis showed that the proportion of poorly differentiated gastric cancer tissues in patients with low expression of LOC339059 was significantly higher than that in patients with increased expression of LOC339059 (the rates of poorly differentiated gastric cancer tissue in patients with low vs. high expression were, respectively, 87.0% and 13.0%, *p* < 0.05).

### 3.4. LOC339059 Suppresses Malignant Phenotypes in GC Cells

**Figure 2:**
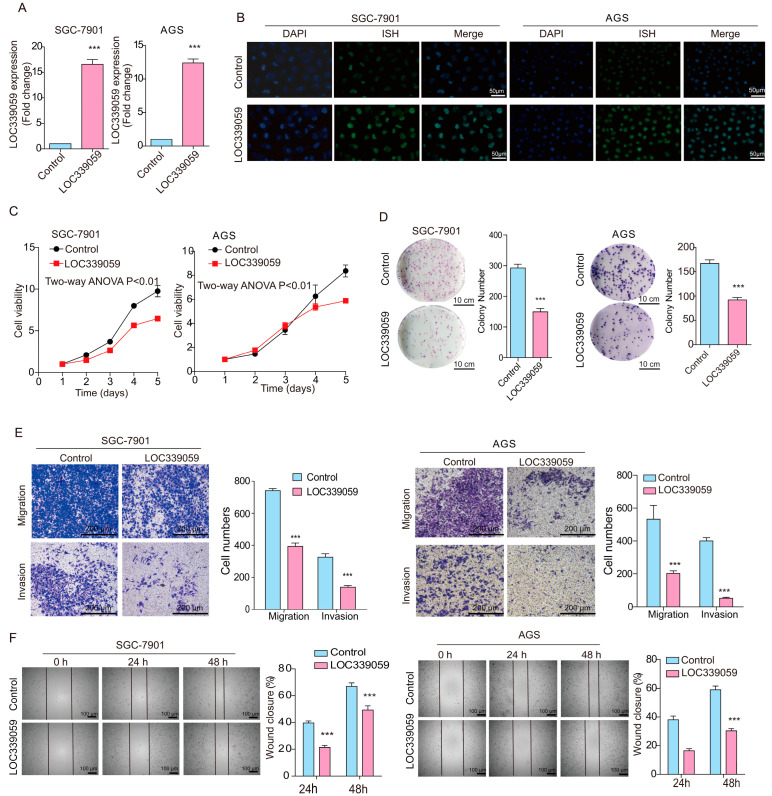
Overexpression of LOC339059 suppresses the growth, migration, and invasion ability of SGC-7901 and AGS cells. (**A**,**B**) qRT-PCR and in situ hybridization validation of the overexpression of LOC339059 via vector transfection. (**C**–**F**) Overexpression of LOC339059 suppressed cell viability as detected using CCK8 (**C**), the clonogenic capacity of the cells as detected via plate clone formation assays (**D**), cell migration and invasion abilities as detected via Transwell assays (**E**), and the cell migration capacity as detected via wound-healing assays (**F**). In (**D**–**F**), the average values of triplicates are presented, and the values in the histogram represent the mean ± SD; *** *p* < 0.001.

To determine the effect of LOC339059 on gastric cancer cells, we established SGC-7901 and AGS gastric cancer cell lines with stable LOC339059 overexpression (Figure 2A). In situ hybridization showed that the expression of LOC339059 was significantly increased in cells, and LOC339059 was mainly located in the cell nucleus (Figure 2B). The cell viability (Figure 2C) and plate clone formation (Figure 2D) were tested to validate the inhibition of proliferation in LOC339059-overexpressing cells. Additionally, cell migration and invasion in vitro were significantly reduced (Figure 2E,F). These results show that the overexpression of LOC339059 inhibited the growth, migration, and invasion of gastric cells in vitro.

### 3.5. LOC339059 Inhibits PDL1 and M2 Macrophage Polarization

**Figure 3:**
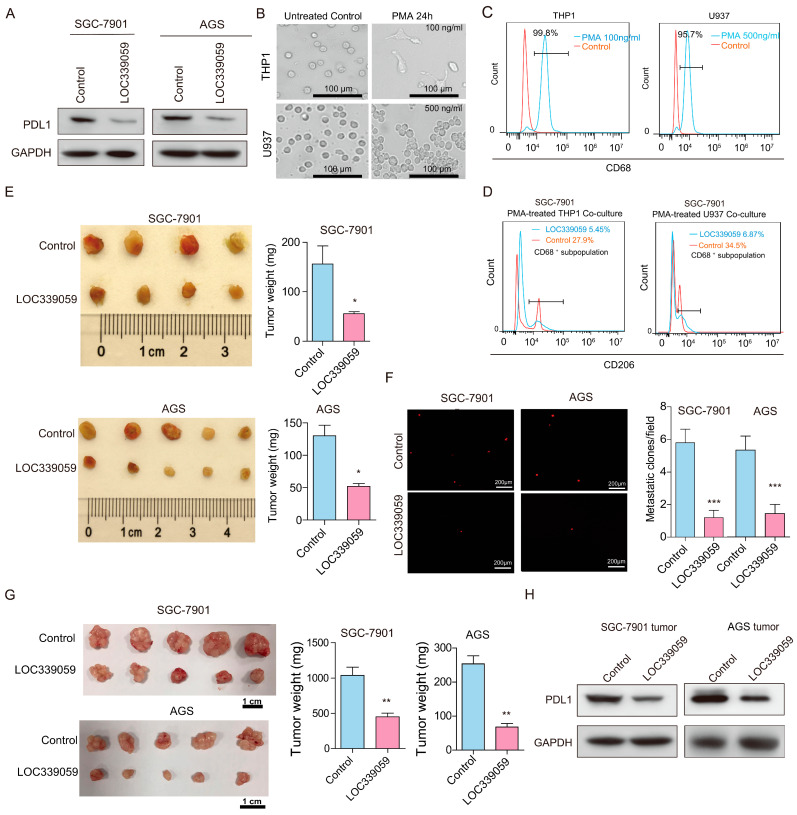
Overexpression of LOC339059 inhibits expression of PDL1; M2 macrophage polarization in vitro; and tumor growth, lung metastasis, and PDL1 expression in vivo. (**A**) PDL1 expression was decreased after overexpression of LOC339059. (**B**) The morphological changes in THP1 and U937 cells after being treated with PMA. (**C**) M0 macrophages were successfully induced after 24 h of PMA treatment and detected via flow cytometry using the CD68 marker. ├┤: range of positive cells. (**D**) Overexpression of LOC339059 in cells induced a larger proportion of M2 macrophages according to flow cytometry analysis of the markers CD68^+^CD206^+^ after the co-culture of tumor cells with M0 macrophages. ├┤: range of positive cells. (**E**,**F**) Overexpression of LOC339059 in cells resulted in decreased tumorigenicity and metastatic foci in chicken embryo lung tissue in vivo, according to CAM assays. (**G**) Overexpression of LOC339059 in cells resulted in decreased tumorigenicity in NOD/SCID mice. (**H**) The expression of PDL1 was down-regulated in tumor tissues in which LOC339059 was overexpressed, according to Western blot analysis. The values in the histogram represent the mean ± SD. *** *p* < 0.001, ** *p* < 0.01, * *p* < 0.05. The uncropped bolts are shown in Supplementary Materials.

Because the PDL1 mRNA level was negatively correlated with LOC339059 in the tumor samples shown in Figure 1D, we further measured the protein levels in SGC-7901 and AGS cells to validate the relationship between PDL1 and LOC339059. PDL1 was also detected to be decreased in cell lines after overexpressing LOC339059 (Figure 3A). To observe the effect of LOC339059 on the polarization of macrophages, we induced the differentiation of THP1 and U937 into M0 macrophages using 100 ng/mL and 500 ng/mL PMA treatment. After 24 h, the morphology of THP1 and U937 cells changed significantly from the original suspended state to adherent growth and pseudopodia (Figure 3B), and the expression ratios for CD68 were 99.8% and 95.7%, respectively (Figure 3C). After the M0 macrophages were co-cultured with vector control cells and LOC339059-overexpressing cells, the CD68^+^/CD206^+^ expression ratio decreased from 27.9% to 5.54% in THP1 cells and from 34.5% to 6.87% in U937 cells (Figure 3D). These results indicate that macrophage M2 polarization may be promoted by co-cultured LOC339059-overexpressing cells, and these cells could further inhibit PDL1 expression in GC tumors.

### 3.6. LOC339059 Inhibits Tumor Growth, PDL1 Expression, and Lung Metastasis In Vivo

Using the chicken embryo allantoic membrane tumorigenesis (CAM) model, Dil-CM-labeled LOC339059-SCG-7901 cells and AGS cells were used to inoculate the CAM for nine days. Tumor growth was measured, and the number of lung metastatic cells was detected using wide-field volumetric microscopes. The tumorigenicity of SCG-7901 and AGS cells was inhibited by LOC339059 (Figure 3E). Moreover, the number of metastatic foci in chicken embryo lung tissue decreased significantly in SCG-7901 and AGS (Figure 3F). The results of the cell-derived xenografted (CDX) NOD/SCID mouse experiment were consistent with those of the CAM model. The weight of the tumors was significantly reduced by LOC339059 (Figure 3G), and the expression level of PDL1 was simultaneously decreased in LOC339059 tumors (Figure 3H). These results indicate that the overexpression of LOC339059 inhibited tumor growth in SCG-7901 and AGS cells in vivo and the expression of PDL1 in tumor tissues.

### 3.7. Interference with LOC339059 Enhanced Malignant Phenotypes, PDL1 Expression, M2 Macrophage Polarization, Tumor Growth, and Metastasis

**Figure 4:**
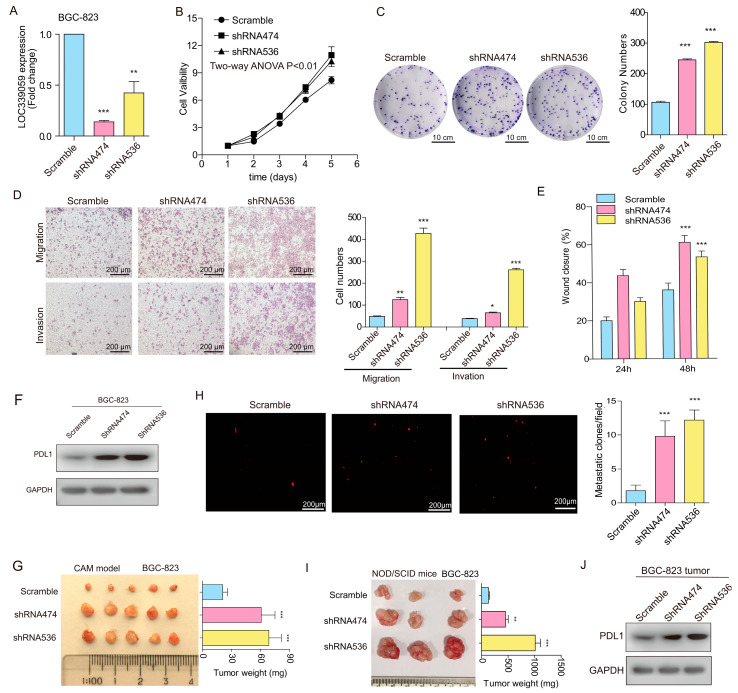
Knockdown of LOC339059 promoted the malignant phenotype in BGC-823 gastric cancer cells. (**A**) qRT-PCR validation of down-regulation of LOC339059 expression by shRNAs. (**B**–**E**) The knockdown of *LOC339059* promoted cell viability (**B**), the clonogenic ability of the cells (**C**), the migration and invasion ability of the cells (**D**), and the wound-healing ability (**E**). (**F**) Knockdown of LOC339059 promoted the expression of PDL1, according to Western blot analysis. (**G**) The cells’ tumorigenicity was increased after the knockdown of LOC339059, according to CAM assays. (**H**) Metastatic foci in chicken embryo lung tissue in vivo, according to CAM assays. (**I**) Tumor growth was increased after the knockdown of LOC339059 in NOD/SCID mice. (**J**) PDL1 expression was increased in mouse tumor tissues after the knockdown of LOC339059, according to Western blot detection. Column charts in (**C**–**E**) represent the mean ± SD from three experiments in triplicate. *** *p* < 0.001, ** *p* < 0.01, * *p* < 0.05. The uncropped bolts are shown in Supplementary Materials.

To conversely clarify the function of LOC339059 in gastric cancer cells, we established a cell line with the stable knockdown of LOC339059. As shown in Figure 4A, both interfering target shRNAs (shRNA 474 and shRNA536) can significantly inhibit the expression of LOC339059 in BGC-823. Silencing LOC339059 increased cell viability, plate colony formation, cell invasion, and migration, as shown in Figure 4B–E. In addition, the expression level of PDL1 was dramatically increased, according to protein testing (Figure 4F). The in vivo experiment using the CAM model further verified that shLOC339059 significantly promoted the tumorigenicity of BCG-823 cells (Figure 4G) and their ability to metastasize to the lungs (Figure 4H). We obtained similar results using the NOD/SCID mouse model (Figure 4I), and the expression of PDL1 in tumor tissues was increased by shLOC339059 (Figure 4J). These results suggest that interference with the presentation of LOC339059 promoted the malignant phenotype in vitro and the expression of PDL1, tumorigenicity, and metastasis in vivo.

### 3.8. High Expression of LOC339059 Decreases Activity of IL-6/STAT3 Signaling Axis

**Figure 5:**
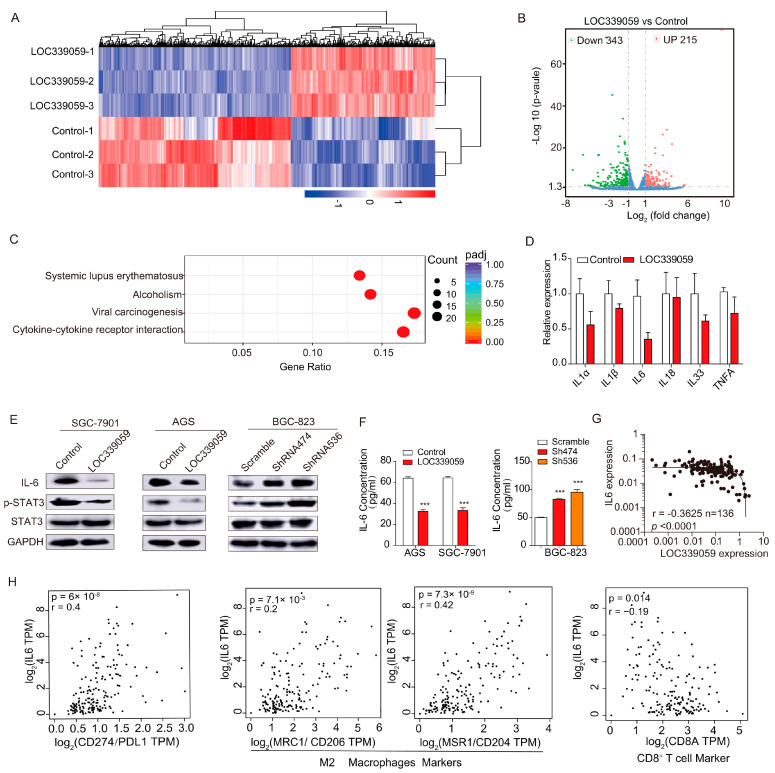
Identification of IL-6/STAT3 signaling associated with LOC339059. (**A**,**B**) Heat and volcano maps of differentially expressed genes (DEGs, differences greater than or equal to 2.0-fold compared with the control group) after overexpression of LOC339059. (**C**) KEGG analysis of enrichment signaling pathways of DEGs associated with LOC339059. (**D**) QRT-PCR detection of the expression levels of cytokines, including interleukin family genes and TNF-a. (**E**) The levels of proteins in the IL-6/STAT3 signaling pathway were suppressed by LOC339059, according to Western blot detection. (**F**) IL-6 secretion in the cell culture supernatant was inhibited by LOC339059, according to ELISA. (**G**) The correlation between IL-6 and LOC339059. (**H**) Linear correlation analysis indicated that IL-6 expression was correlated with the expression of immune molecules (PDL1, M2 macrophages, and T cells). Values in the histogram represent the mean ± SD. *** *p* < 0.001. The uncropped bolts are shown in Supplementary Materials.

To further explore the molecular mechanism by which LOC339059 inhibited the malignant biological behavior of gastric cancer cells, we performed an RNA sequencing analysis of differentially expressed genes (DEGs) in parental vector control and overexpression cell lines (Figure 5A). The results showed 558 DEGs with multiple differences greater than 2, of which 215 were up-regulated and 323 were down-regulated (Figure 5B). KEGG signaling pathway analysis showed that the signaling pathways were focused on alcoholism, systemic lupus erythematosus, viral carcinogenesis, and cytokine–cytokine-receptor interaction (Figure 5C, *p* < 0.05). The expression of the cytokines IL-1A, IL-1B, IL-6, and IL-33 was significantly decreased according to q-PCR verification (Figure 5D). We then verified the changes in the levels of proteins involved in IL-6/STAT3 signaling in the cells with LOC339059 overexpression or interference using Western blotting. Consistent with expectations, the IL-6 and p-STAT3 protein expression decreased after the overexpression of LOC339059 and, conversely, increased after interference with LOC339059 expression (Figure 5E). IL-6 secretion was decreased in the supernatant of LOC339059-overexpressing cells according to the results obtained using an ELISA detection kit. Furthermore, an increase in IL-6 in the supernatant of shLOC339059 BCG-823 cells was detected (Figure 5F). In addition, a negative correlation between the expression of LOC339059 and IL-6 in gastric tumor tissues was detected via qPCR in a total of 136 GC samples (Figure 5G, Pearson correlation r = −0.3625, *n* = 136, *p* < 0.0001). GEPIA (http://gepia.cancer-pku.cn/, accessed on 5 November 2022) analysis using Stomach data in the GTEx database suggested that the expression level of IL-6 was positively correlated with PDL1 expression (Pearson correlation r = 0.4), the M2 macrophage biomarker CD206 (Pearson correlation r = 0.2), and CD204 (Pearson correlation r = 0.42) but negatively correlated with the CD8-positive T-cell marker CD8A (Pearson correlation r = −0.19) (Figure 5H), suggesting that IL-6 participates in immune regulation in gastric cancer. Based on these results, we believe that LOC339059 can inhibit IL-6/STAT3-signaling-mediated PDL1 expression and M2 polarization. 

### 3.9. IL-6 Rescue Partly Reversed the Inhibitory Effect of LOC339059 on the Malignant Phenotype, PDL1 Expression, and M2 Macrophage Polarization

**Figure 6:**
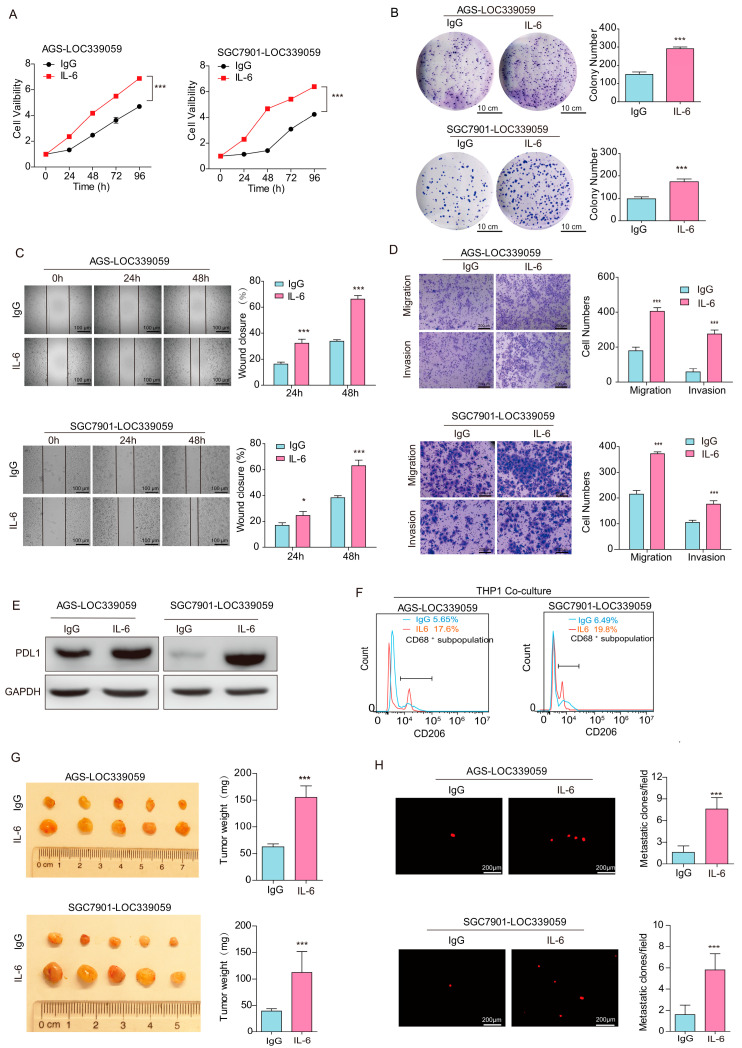
Rescue of IL-6 protein weakened the inhibitory effect of LOC339059 on the malignant phenotype of BGC-823 gastric cancer cells. Supplementing with IL-6 protein in LOC339059-overexpressing cells not only restored the cell viability (**A**), cell cloning ability (**B**), wound-healing ability (**C**), and cell migration and invasion abilities (**D**) but also increased the PDL1 expression (**E**) and M2 macrophage percentage according to the CD68^+^CD206^+^ ratio, determined using a flow cytometer on tumor cells co-cultured with M0 macrophages (**F**). Furthermore, restoring the IL-6 protein in LOC339059-overexpressing cells enhanced tumor growth (**G**) and lung metastasis (**H**), according to CAM assays. The weight of the tumor is indicated on the right side of G. The (**H**) panel shows the detection of metastasis in chicken embryo lung tissue via laser confocal microscopy, and the bar chart on the right side of (**H**) represents the number of chicken embryo lung metastases (>10 cells) in different groups of cells. The (**B**–**D**) experiments were repeated three times, and the bars represent the mean ± SD. *** *p* < 0.001, * *p* < 0.05. The uncropped bolts are shown in Supplementary Materials.

To verify the role of IL-6 in the inhibitory effect of LOC339059 on the malignant phenotype, we conducted a functional experiment with exogenous IL-6 (final concentration: 25 ng/mL) supplementation. Compared to the IgG control cells, rescuing IL-6 in the LOC339059-overexpressing AGS and SGC-7901 cells increased the cell viability (Figure 6A), enhanced the colony formation ability (Figure 6B), and increased the cell migration and invasion abilities (Figure 6C,D). Consistent with the literature, exogenous IL-6 supplementation in LOC339059 cells could partially restore the PDL1 expression in tumor cells (Figure 6E). IL-6 partially eliminated LOC339059′s inhibition of the polarization of macrophages. CD206^+^ subsets recovered 17.6–19.8% of cells (almost 3–4-fold vs. the IgG group) of the total CD68^+^ THP-1 (Figure 6F). In the CAM model, following treatment with exogenous human IL-6, the tumorigenicity of LOC339059-overexpressing AGS and SGC-7901 cells was enhanced (Figure 6G). The number of lung metastatic cells was significantly increased (Figure 6H). These results demonstrate that rescuing IL-6 expression partially attenuates the inhibitory effect of LOC339059 on the malignant phenotype, cell PDL1 expression, and macrophage M2 polarization. That is, LOC339059 plays an inhibitory role in influencing the malignant phenotype of gastric cancer, at least partially by down-regulating IL-6.

### 3.10. LOC339059 Competitively Interacted with c-Myc, Resulting in Reduced Transcriptional Activation of IL-6

**Figure 7:**
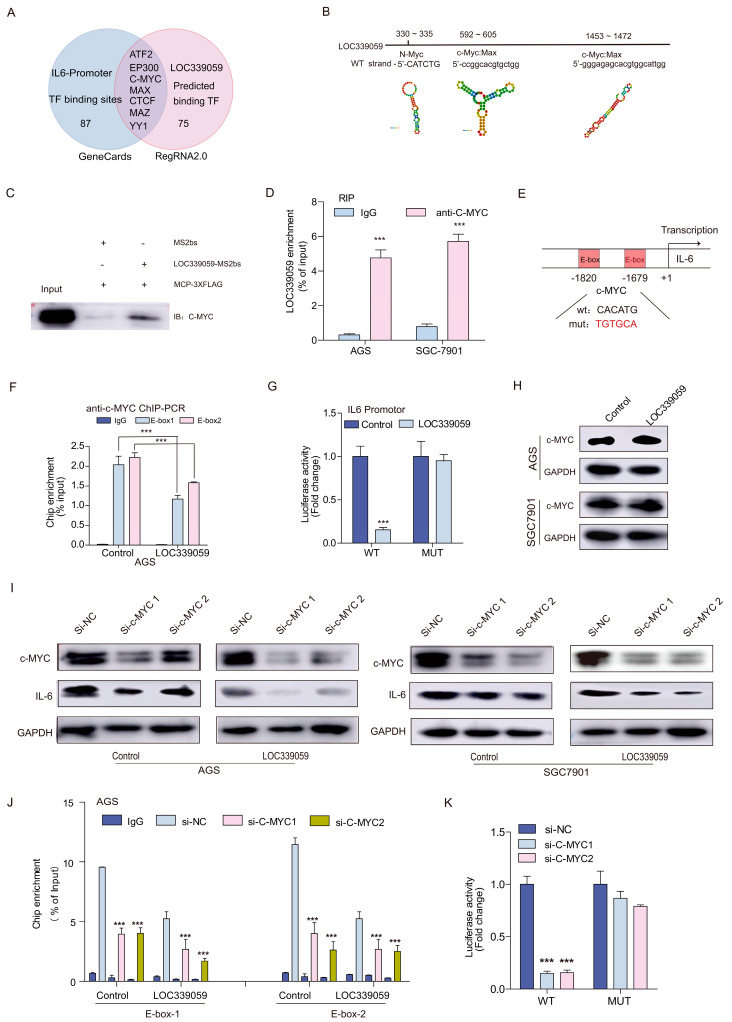
Competitive binding of LOC339059 with c-Myc reduced the effect of c-Myc on IL-6 transcription activation. (**A**) Bioinformatics predicted seven candidate transcription factors involved in regulating IL-6 expression associated with LOC339059. (**B**) RegRNA2.0 predicted two c-Myc binding sites in LOC339059 RNA. (**C**) RNA pull-down indicated the interaction of c-Myc with LOC339059. (**D**) RIP combined with qRT-PCR detected the enrichment of the c-Myc protein on LOC339059 RNA. (**E**) JASPAR predicted two binding sites (E-box) for c-Myc within 2 kb upstream of the IL-6 promoter. (**F**) ChIP-PCR detected less enrichment of c-Myc on E-box1 and E-box2 of the IL-6 promoter in LOC339059-overexpressing cells compared with control cells. (**G**) Promoter luciferase reporter assay detected the inhibitory effect of LOC339059 on the transcriptional activity of wild-type IL-6 but not the mutation one. (**H**) Western blotting detected that c-Myc proteins were not altered after LOC339059 overexpression. (**I**) Knockdown of c-Myc expression suppressed IL-6 expression, as shown via Western blotting. (**J**) Knockdown of c-Myc expression decreased the enrichment of c-Myc at IL-6 promoters, according to ChIP-PCR detection. (**K**) A promoter luciferase reporter assay suggested that the knockdown of c-Myc suppressed IL-6 transcriptional activity. *** *p* < 0.001. The uncropped bolts are shown in Supplementary Materials.

The nuclear location of LOC339059 suggests that it functions in the regulation of gene transcription. To determine how LOC339059 regulates the expression of IL-6, we intersected the candidate transcription factors binding to the IL-6 promoter with Gencards and predicted LOC339059-binding transcription factors using RegRNA2.0 software. Figure 7A shows that seven candidate transcription factors (ATF2, EP300, c-Myc, MAX, CTCF, MAZ, and YY1) were screened out. RegRNA2.0 predicted two c-Myc binding sites in LOC339059 RNA (Figure 7B). The interaction between LOC339059 and c-Myc was further proven using an RNA pull-down assay (Figure 7C) and RNA immunoprecipitation (RIP) assay (Figure 7D). We obtained two c-Myc binding sites from the JASPAR database (http://jaspar.genereg.net, accessed on 5 November 2022), namely, E-box1 and E-box2, within 2 kb upstream of the IL-6 promoter (Figure 7E). The ChIP-PCR experiment using the c-Myc antibody showed decreased c-Myc binding to the two binding sites of AGS-LOC339059 (Figure 7F). A 2 kb sequence of the IL-6 promoter was cloned into the upstream part of the luciferase gene of the PGL3-basic plasmid. The results of the fluorescence double-reporter experiment showed that LOC339059 could inhibit the transcription activity of the IL-6 promoter (Figure 7G). Since LOC339059 has no effect on the expression level of c-Myc at the protein level (Figure 7H) and has no predicted binding sites in the IL-6 promoter, we propose the hypothesis that LOC339059 binds competitively to c-Myc, resulting in reduced c-Myc enrichment at the IL-6 promoter. We conducted interference experiments with regard to c-Myc to verify whether c-Myc activated IL-6 transcription. After interference with c-Myc expression, the expression level of IL-6 decreased in the control cells but more obviously decreased in LOC339059-overexpressing cells (Figure 7I). The ChIP-PCR results showed that the enrichment of c-Myc at the IL-6 promoter decreased after interfering with c-Myc expression (Figure 7J), and the fluorescence double-reporter results showed that LOC339059 could inhibit the transcriptional activity of the wild-type IL-6 promoter, but not the mutation type (Figure 7K). These results indicate that LOC339059 reduced the c-Myc-mediated transcriptional activation of IL-6 by competitively binding to c-Myc. 

**Figure 8:**
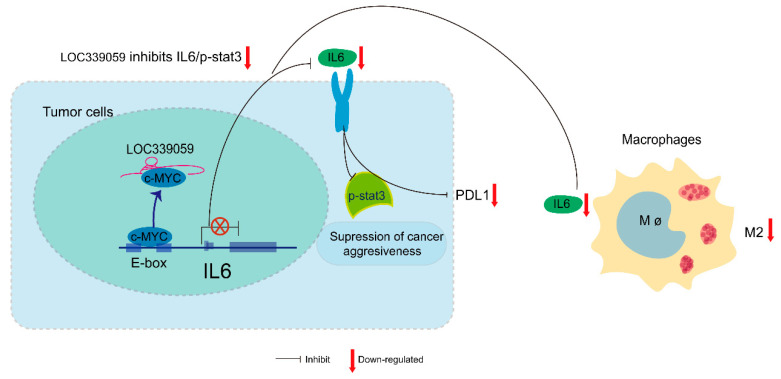
A hypothetical pattern of the competitive binding of LOC339059 to c-Myc, resulting in less activation of IL-6 transcription, followed by the inhibition of STAT3 signaling inhibition, and, therefore, decreased PDL1 expression and M2 macrophage polarization.

All of the results suggest that LOC339059 may function as a tumor suppressor via the inhibition of IL-6/STAT3 signaling through endogenous competition with c-Myc, competitively inhibiting the latter’s binding to the IL-6 promoter, resulting in the decreased transcription of IL-6 and, therefore, lower PDL1 expression and M2 macrophage polarization (Figure 8).

## 4. Discussion

Based on our previous study, we searched lncRNAs in the GEO database for their possible involvement in gastric cancer. We found substantially lower LOC339059 expression in tumors than in adjacent tissues [25]. Our cohort analysis further indicated LOC339059′s typical expression in gastric cancer tissues in this study. In addition, patients with high LOC339059 exposure had favorable overall survival times after surgery. More importantly, LOC339059 was negatively correlated with immune checkpoint molecules (PDL1 and CTLA4) and CD204, CD206, and CD163. The potential role of LOC339059 in the regulation of the immune microenvironment in gastric cancer in response to M2 macrophages is nearly identical to the roles of CD8+T, Treg, NK, and other immune-infiltrating cells and more significant than the role of M1 macrophages. Lower LOC339059 expression in gastric cancer was associated with poorer differentiation statuses in tumors. This finding suggests that LOC339059 may be a tumor suppressor involved in the regulation of the immune microenvironment in gastric cancer, potentially serving as a biomarker for cancer prognosis.

Apart from one original article [23] and one review [24] referring to LOC339059, our findings offer new information on the role of the lncRNA LOC339059 in gastric cancer. In 2021, Yang et al. first reported that LOC339059 was down-regulated and had remarkable diagnostic value for papillary thyroid carcinoma (PTC) patients and predicted the crucial role of LOC339059-STK32A-hsa-miR-199b-5p in PTC based on the analysis of RNA sequencing data from The Cancer Genome Atlas (TCGA) database [23]. However, the results lacked support from a clinical validation cohort of PTC patients. In this study, we identified the down-regulation of LOC339059 in GC tissues compared to normal tissues based on microarray data from the GEO database, consistent with the results for LOC339059 in tumors. Moreover, we validated the above results with our cohort. We found that high LOC339059 expression was associated with a favorable overall survival time after surgery and was associated with the good differentiation of tumor cells. The mechanism of the down-regulation of LOC339059 might involve promoter methylation or other related forms of transcriptional regulation, which is still not understood. It is known that the excellent differentiation of tumor cells is often associated with a better prognosis [27]. However, whether the tumor-suppressor gene LOC339059 promotes the differentiation of cancer cells still requires more experiments to validate its effect on the molecular differentiation markers b-catenin, CD44, and Cyclin D1 [27]. Besides the consistent findings of low LOC339059 in cancer sites, we also extensively studied the underlying molecular mechanisms. 

The gene encoding LOC339059 is on chromosome 16 (16q24.2) and is 2137 nucleotide bases in length. The LOC339059 gene encodes one of the intronic lncRNAs [28] and is located in the intron of the Piezo1 gene, but LOC339059 and Piezo1 are transcribed in opposite directions. Whether there is any relationship between LOC339059 and the Piezo1 gene is still unclear.

To explore the cellular functions and signaling pathways of LOC339059, we performed gain-of-function and loss-of-function experiments and RNA sequencing for vector control and stable overexpression cells, respectively. For the first time, LOC339059 was identified as a tumor-suppressor gene that can suppress the malignant phenotypes of gastric cancer cells both in vitro and in vivo. Notably, we also determined the inactivation effect of LOC339059 on PDL1 expression and M2 macrophage biomarkers through both in vitro experiments and the finding of negative correlations between gene expression levels in vivo, suggesting a crucial role for LOC339059 in regulating immune checkpoint molecules and tumor-infiltrating cells. Interestingly, our bioinformatic investigation identified four enrichment signaling pathways: alcoholism, systemic lupus erythematosus, viral carcinogenesis, and cytokine–cytokine-receptor interaction. Further validation confirmed the decreased expression of the cytokines IL1A, IL1B, IL-6, and IL33. We focused on IL-6/STAT3 based on previous reports of such signaling in the activation of PDL1 expression and M2-type macrophages [29,30]. Consistent with expectations, we found similar changes in opposite directions of activity; that is, overexpression led to the down-regulation of IL-6/STAT3 signaling, whereas upon LOC339059 knockdown, IL-6/STAT3 signaling was up-regulated. In addition, we found a negative association between LOC339059 and IL-6 expression in cancer tissues, suggesting an interrelation between them inside human tumor tissues. In addition to a previous study referring to IL-6/STAT3 signaling in the activation of PDL1 expression in lung cancer [29] and M2-type macrophages in hepatocellular carcinoma [30], here, we provide indirect and direct evidence for such a function of IL-6 in gastric cancer. Using the GTEx database, the expression level of IL-6 was positively correlated with PDL1 expression and M2 macrophage biomarkers, namely, CD206 and CD204, indirectly supporting the idea that IL-6 regulates immune function in gastric cancer. IL-6 rescue experiments provided direct evidence for the activation of PDL1 and M0-to-M2 polarization. However, in vivo IL-6 rescue experiments still need to be carried out. 

LncRNAs’ functions depend on their cellular location. The function of one nuclear lncRNA is believed to be achieved through transcriptional regulation via indirect or direct binding to transcriptional factors or to a genomic DNA sequence [31]. For example, during the last few years, a nuclear lncRNA called NEAT1 (Nuclear-Enriched Abundant Transcript 1) has recently gained considerable attention due to its important contributions to gene regulation through NEAT1-binding proteins, such as enzymes, transcription factors, and receptors [32,33]. RegRNA 2.0 [34] is an integrated web server for identifying functional RNA motifs in an input sequence, which is often used for predicting the potential binding of lncRNAs to transcriptional factors or microRNAs [35]. Here, we first identified that LOC339059 is a nuclear lncRNA via ISH, regarded as the gold-standard approach [36], in fixed cells and tumor tissues and then predicted potential binding factors using RegRNA 2.0. Focusing on the downstream regulation of IL-6, we narrowed the candidates down to seven candidates through the intersection of the potential binding factors with the predicted binding transcriptional factors for the IL-6 promoter. An experiment validated the binding of LOC339059 to the c-Myc protein and the transactivation of IL-6 by c-Myc, which is consistent with previous direct evidence that IL-6 expression is activated by the transcription factor c-Myc [37]. Since cytoplasmic lncRNAs can act as gene regulators via the sponging of cytosolic factors or miRNAs [38,39], the idea that the nuclear lncRNA LOC339059 can also function as a c-Myc sponge to indirectly regulate the transcriptional activation of IL-6 is certainly reasonable. Similar competing mechanisms have previously been verified. For example, Elguindy et al. reported that the lncRNA NORAD can outcompete thousands of other transcripts to bind PUM proteins, enabling it to act as a potent PUM inhibitor [40]. However, whether and how it forms ribonucleoprotein (RNP) granules in the regulation of IL-6 expression needs further study.

## 5. Conclusions

Our study showed that the low expression of LOC339059 was associated with the poor survival of patients and differentiation of tumor cells in gastric cancer. The interaction of nucleus-localized LOC339059 with c-Myc inhibited the latter’s ability to promote IL-6 transcription, thereby inhibiting IL-6/STAT3-mediated PDL1 expression and M2 polarization. 

## Figures and Tables

**Table 1:** Analysis of LOC339059 expression and clinical characteristics in gastric cancer.

Characteristics	High Expression	Low Expression	*p* Value
	*n*	*n*	
Age (years)			0.728
>60	26 (36.10%)	46 (63.9%)	
<60	24 (32.4%)	50 (67.60%)	
Gender			1.000
Male	36 (34.0%)	70 (66.0%)	
Female	14 (35.0%)	26 (65.0%)	
Size			0.298
<5 cm	29 (37.2%)	49 (62.8%)	
>5 cm	19 (29.2%)	46 (70.8%)	
Differentiation			0.029 *
Good	47 (38.2%)	76 (61.8%)	
Poor	3 (13.0%)	20 (87.0%)	
Invasion depth			0.051
T1/T2	3 (15.0%)	17 (85.0%)	
T3/T4	47 (37.3%)	79 (62.7%)	
Tumor embolus			0.723
Yes	30 (61.2%)	55 (57.3%)	
No	19 (38.8%)	41 (42.7%)	
Lymphatic metastasis			0.126
Negative	6 (22.2%)	21 (77.8%)	
Positive	44 (37.0%)	75 (63.0%)	
Distant metastasis			0.445
Yes	17 (39.5%)	26 (60.5%)	
No	33 (32.0%)	70 (68.0%)	

Note: * *p* < 0.05.

## Data Availability

All data included in this manuscript are available upon noncommercial request.

## Supplementary Materials

The following supporting information can be downloaded at https://www.mdpi.com/article/10.3390/cancers15225313/s1. Figure S1. Linear correlation analysis of the correlation between LOC339059 expression and the expression of immune molecular markers in tumors.
