# Supplementary figures and images for: Long Non-Coding RNA LOC339059 Attenuates IL-6/STAT3-Signaling-Mediated PDL1 Expression and Macrophage M2 Polarization by Interacting with c-Myc in Gastric Cancer
Source: Cancers (Basel). 2023 Nov 7;15(22):5313. doi: 10.3390/cancers15225313 (PMC10670112; doi:10.3390/cancers15225313)

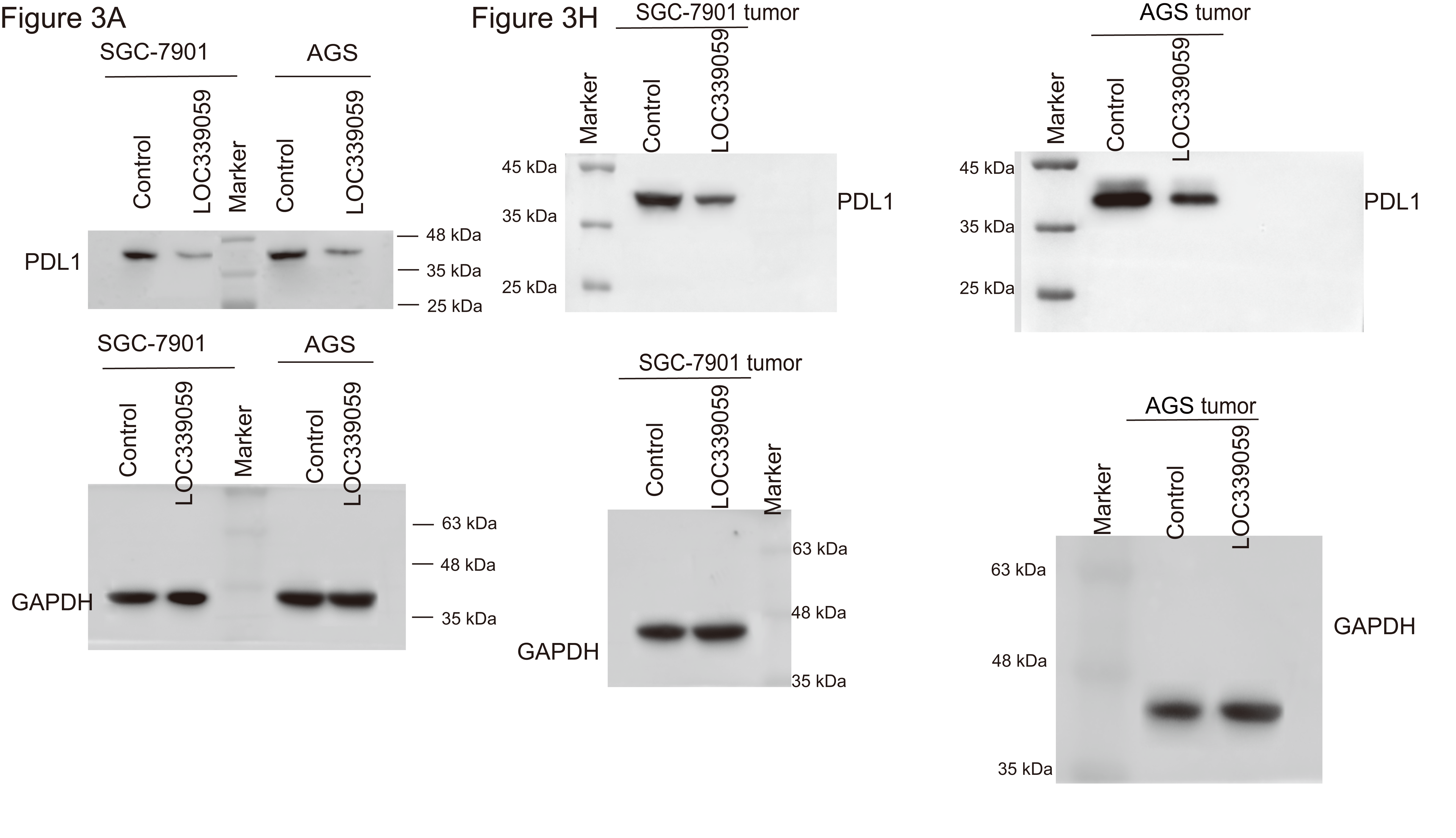

Supplement: Supplementary file 1 [file cancers-15-05313-s001.zip › WB original-Fig3A-3H.tif]

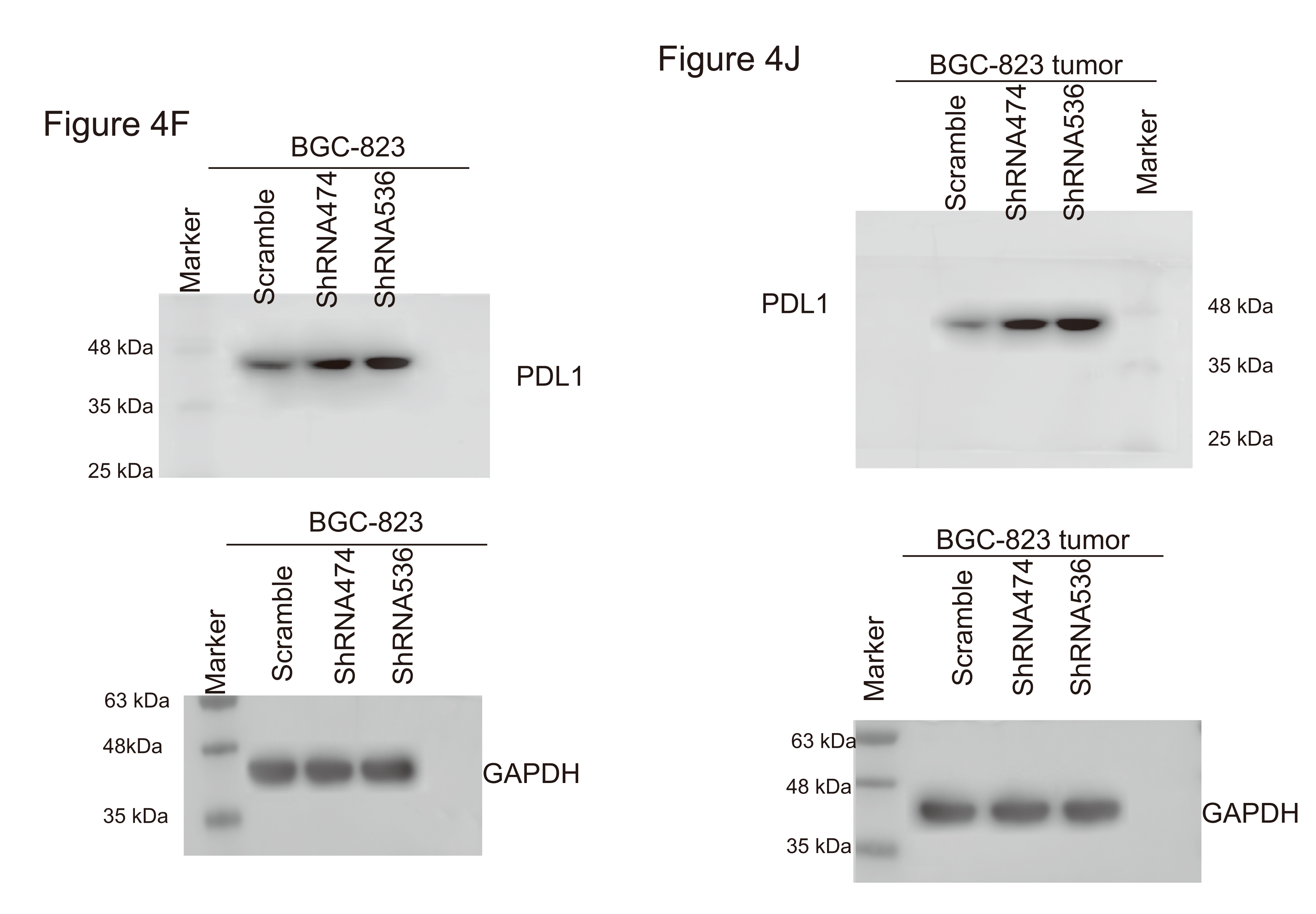

Supplement: Supplementary file 1 [file cancers-15-05313-s001.zip › WB original-Fig4F-4J.tif]

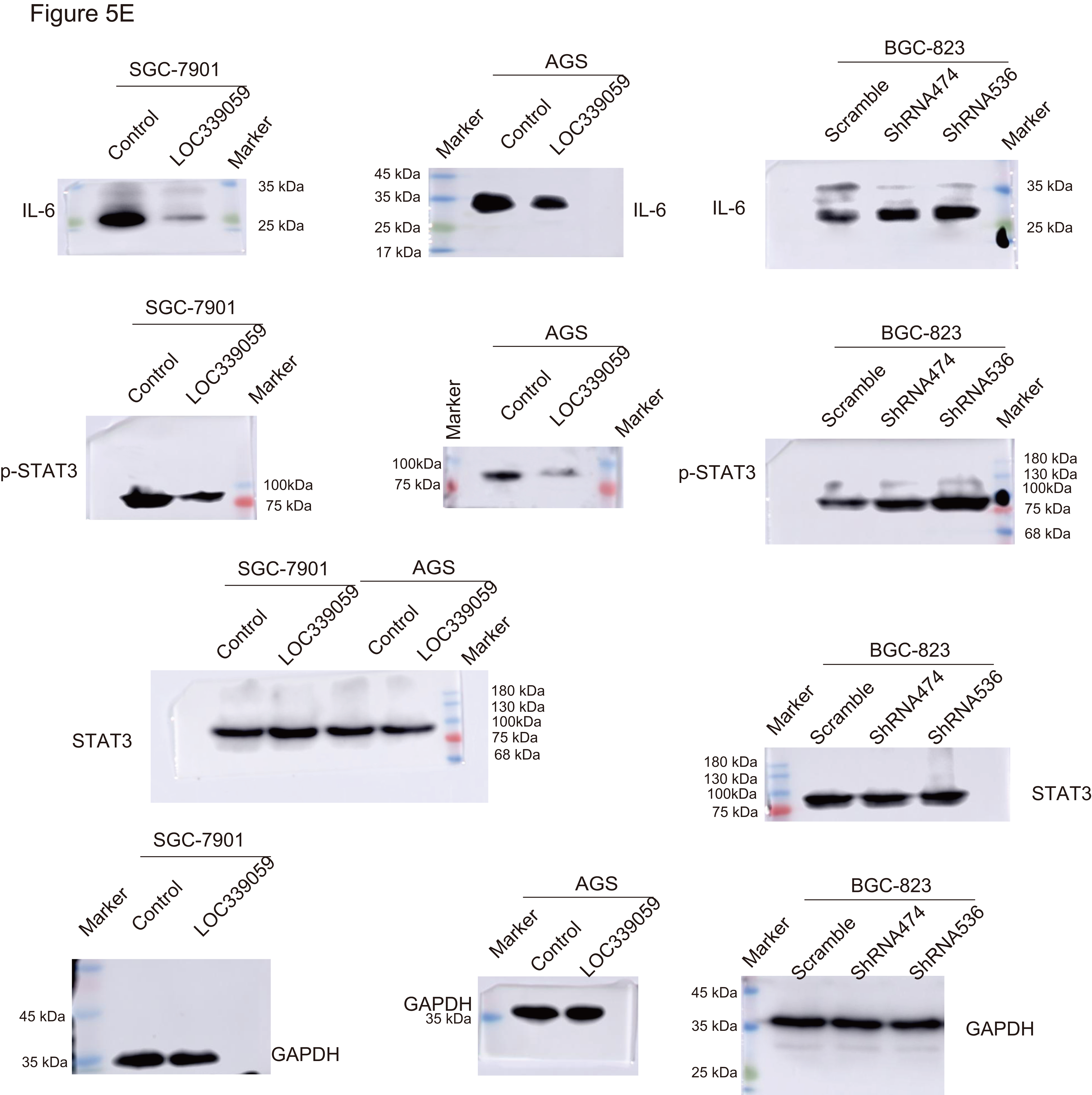

Supplement: Supplementary file 1 [file cancers-15-05313-s001.zip › WB original-Fig5E.tif]

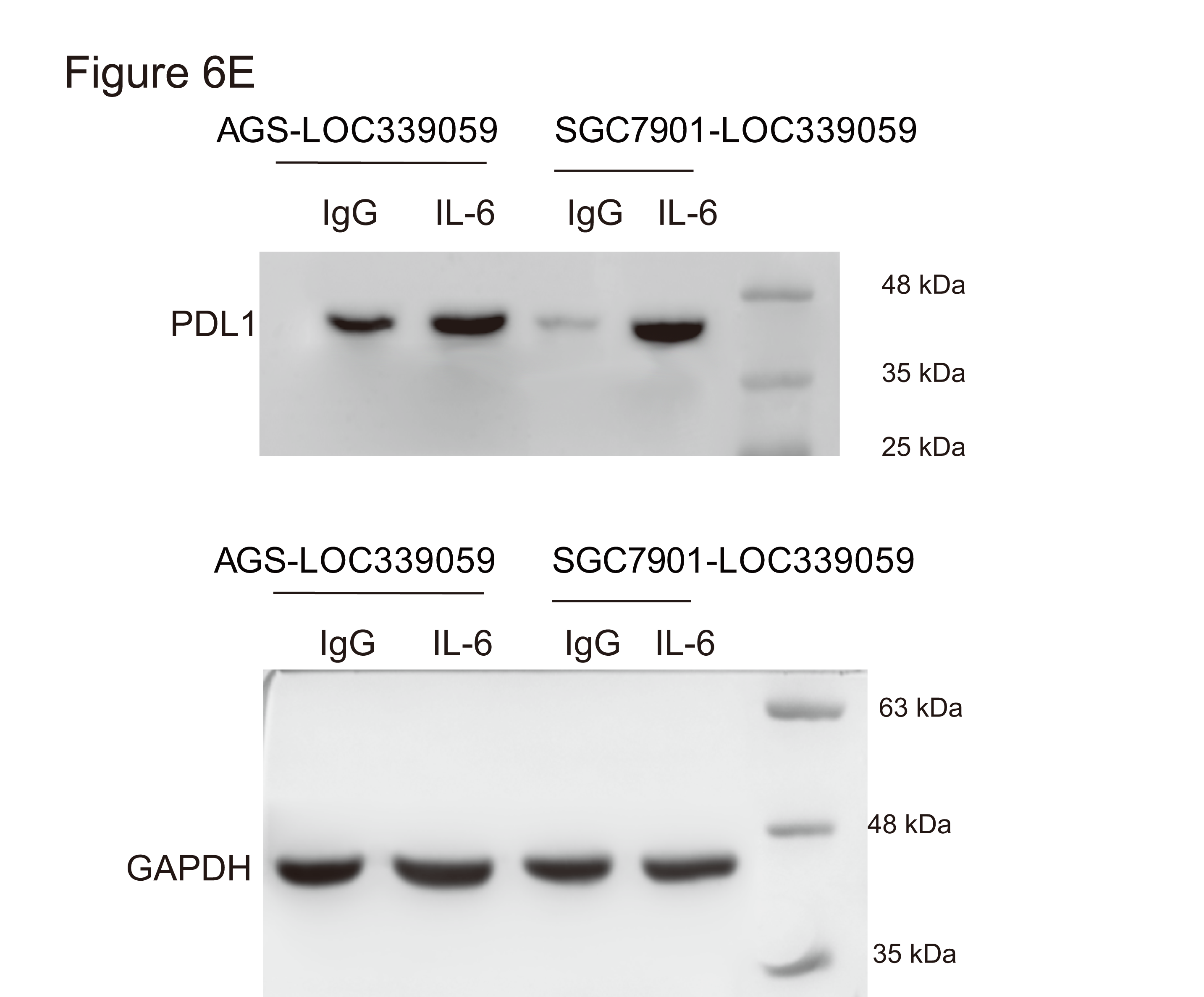

Supplement: Supplementary file 1 [file cancers-15-05313-s001.zip › WB original-Fig6E.tif]

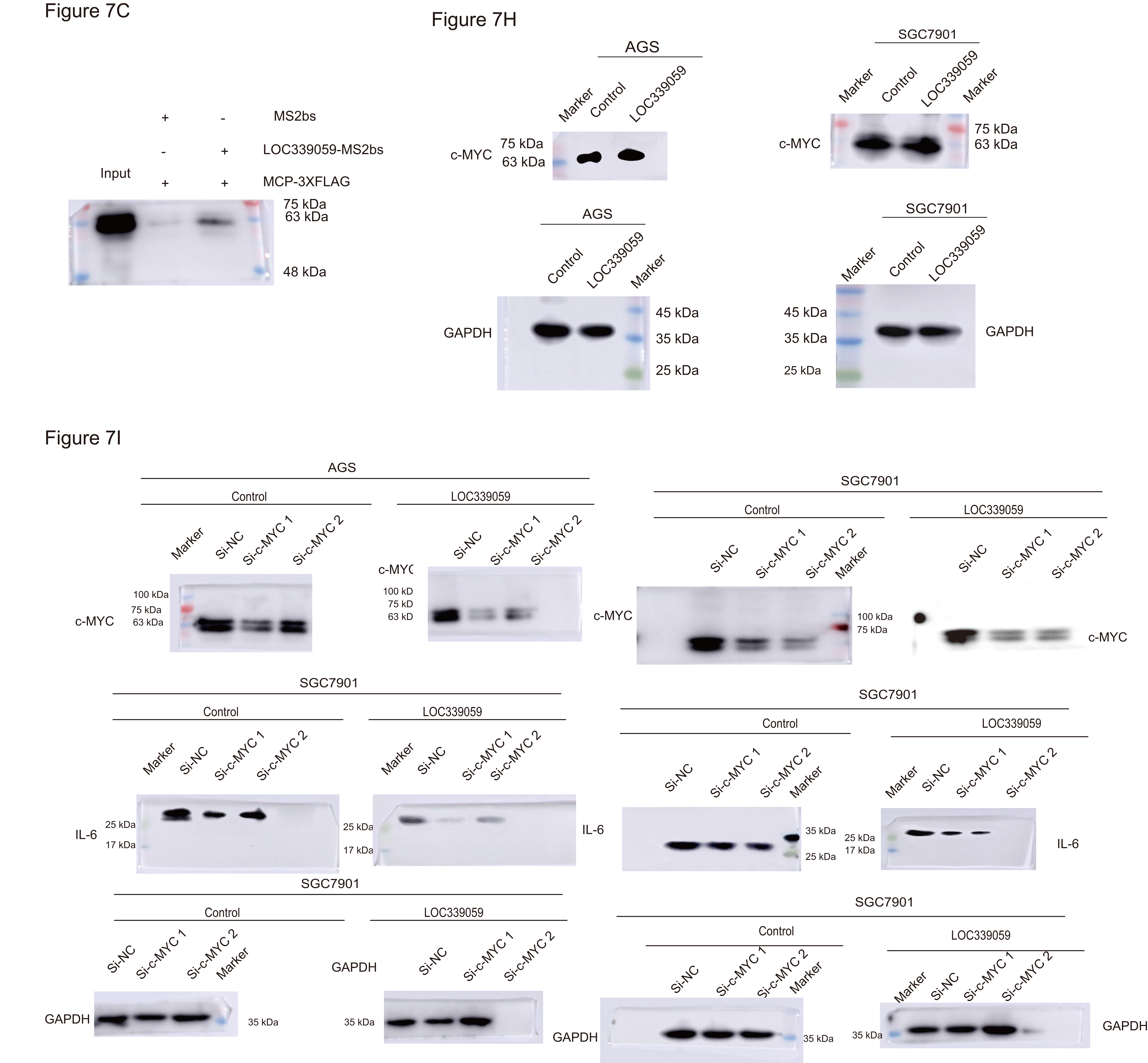

Supplement: Supplementary file 1 [file cancers-15-05313-s001.zip › WB original-Fig7C-7H-7I.tif]

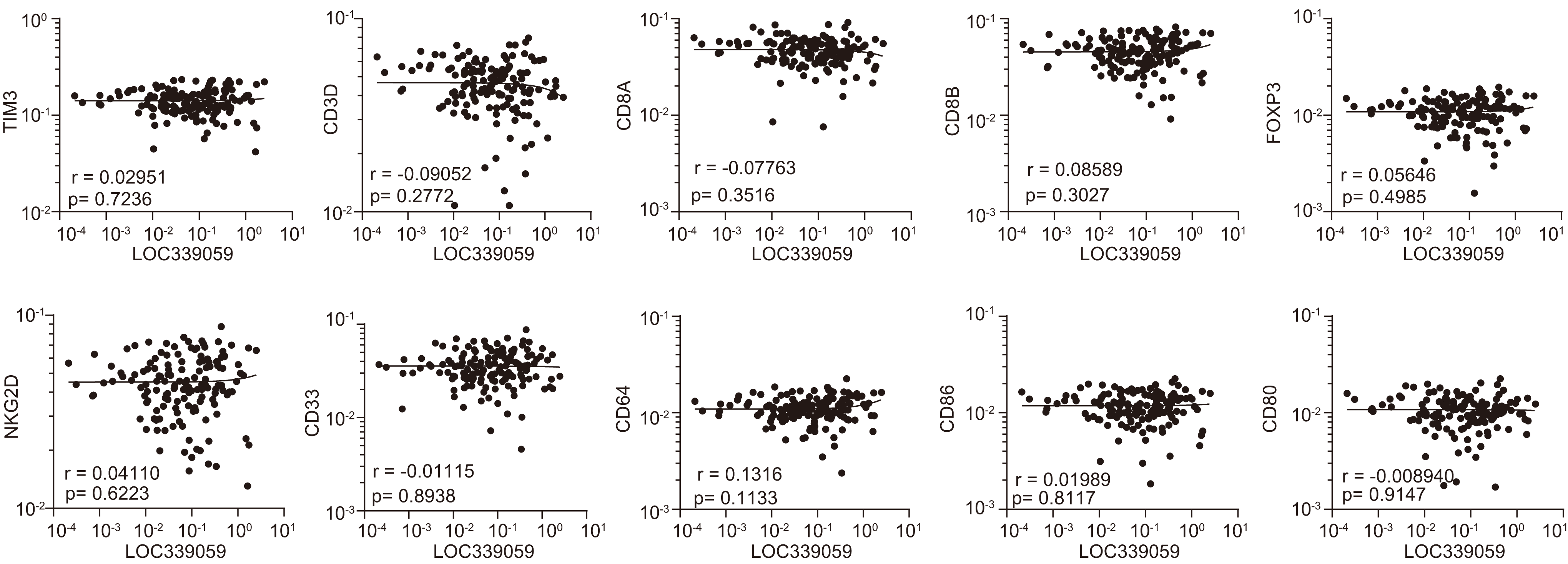

Supplement: Supplementary file 1 [file cancers-15-05313-s001.zip › Supple-fig1.tif]
